# Supplementary material for: Histidine modulates amyloid-like assembly of peptide nanomaterials and confers enzyme-like activity
Source: Nat Commun. 2023 Sep 19;14:5808. doi: 10.1038/s41467-023-41591-1 (PMC10509148; doi:10.1038/s41467-023-41591-1)
Supplement: Supplementary file 1 — Supplementary Information [file 41467_2023_41591_MOESM1_ESM.pdf]

Histidine modulates amyloid-like assembly of peptide nanomaterials and confers enzyme-like activity

Ye Yuan[1, 2, 3], Lei Chen[2], Lingfei Kong[4], Lingling Qiu[5], Zhendong, Fu[1], Minmin Su[2], Yuan Liu[3], Miaomiao Cheng[3], Saiyu Ma[3], Xiaonan Wang[2], Changhui Zhao[1], Jing Jiang[2], Xinzheng Zhang[4], Liping Wang [1*], Lizeng Gao[2, 3*]

[1]Key Laboratory for Molecular Enzymology and Engineering, School of Life Sciences, Jilin University, Changchun 130012, China

[2]CAS Engineering Laboratory for Nanozyme, Key Laboratory of Biomacromolecules, Institute of Biophysics, Chinese Academy of Sciences, Beijing 100101, China

[3]Nanozyme Medical Center, School of Basic Medical Sciences, Zhengzhou University, Zhengzhou 450001, China

[4]National Laboratory of Biomacromolecules, CAS Center for Excellence in Biomacromolecules, Institute of Biophysics, Chinese Academy of Sciences, Beijing 100101, China

[5]Key Laboratory of Animal Genetics and Breeding and Molecular Design of Jiangsu Province, Yangzhou University, Yangzhou, China

Supplementary Figs. 1-33

Supplementary Tables 1-3

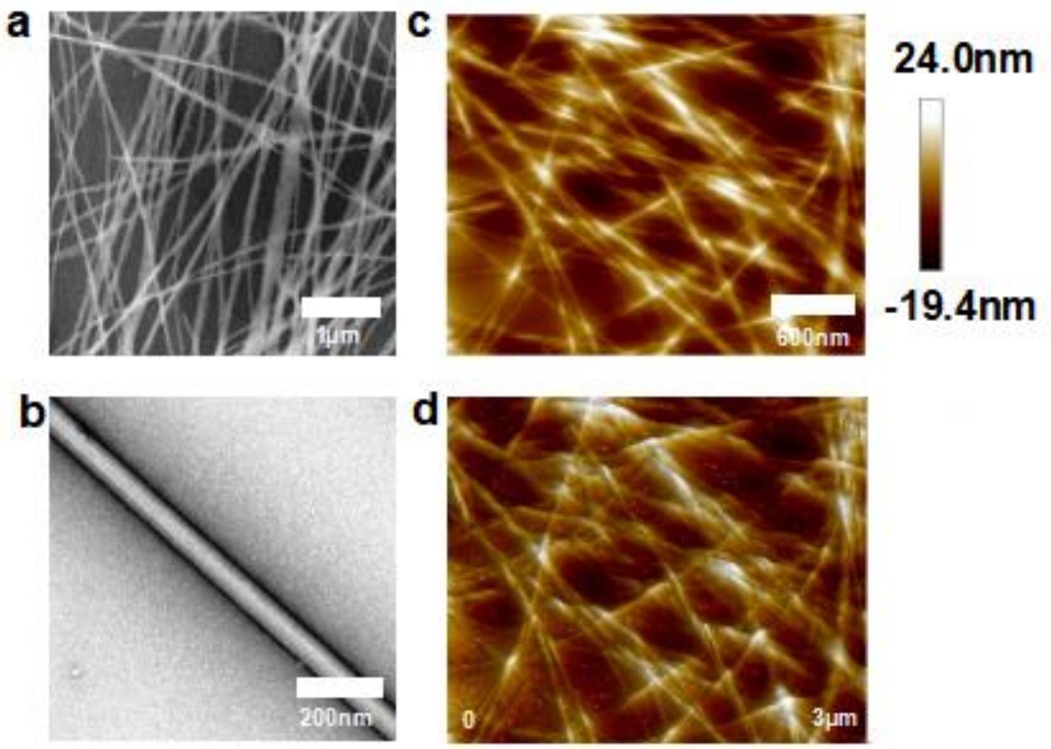

Supplementary Fig. 1 Basic characterizations for Fmoc-F-F (His). a HAADF for staggered net-like structure Fmoc-F-F (His). **b** Negative staining TEM for the characterization of single filament. **c** AFM for Fmoc-F-F (His) filaments. **d** Phase diagram of Fmoc-F-F (His) filaments characterized by AFM. Three times each experiment was repeated independently with similar results. Representative images are shown.

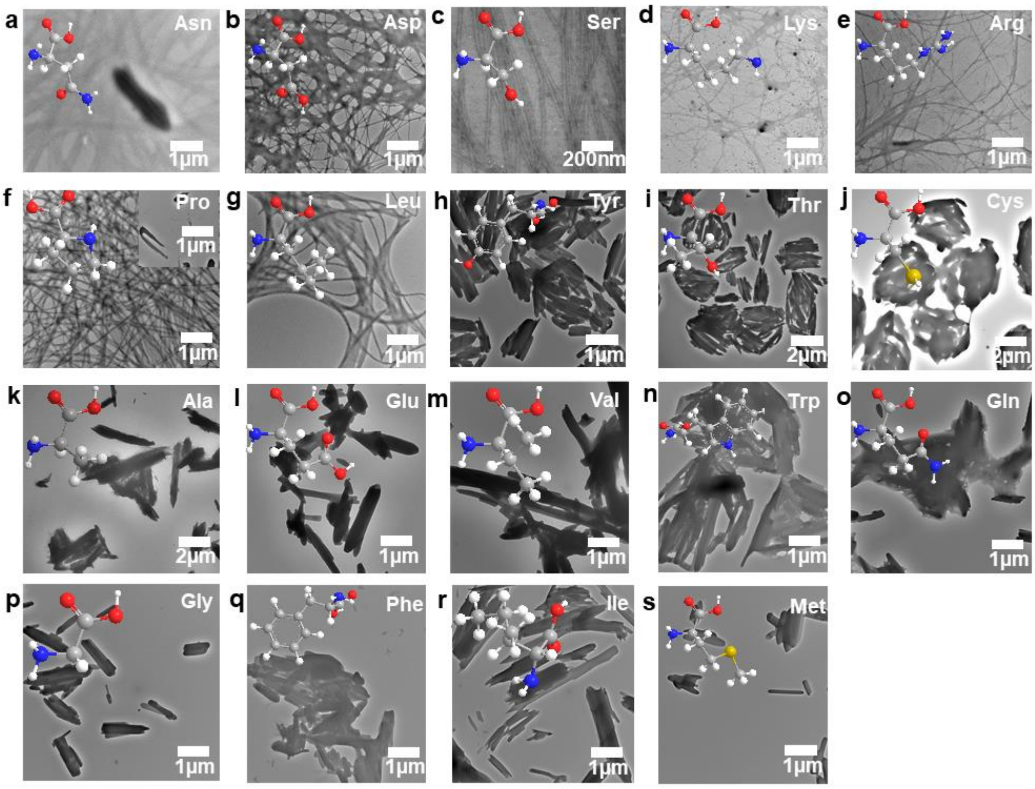

Supplementary Fig. 2 TEM characterization for Fmoc-F-F co-assembled with different amino acids in water. **a** Fmoc-F-F (Asn). **b** Fmoc-F-F (Asp). **c** Fmoc-F-F (Ser). **d** Fmoc-F-F (Lys). **e** Fmoc-F-F (Arg). **f** Fmoc-F-F (Pro). **g** Fmoc-F-F (Leu). **h** Fmoc-F-F (Tyr). **i** Fmoc-F-F (Thr). **j** Fmoc-F-F (Cys). **k** Fmoc-F-F (Ala). **l** Fmoc-F-F (Glu). **m** Fmoc-F-F (Val). **n** Fmoc-F-F (Trp). **o** Fmoc-F-F (Gln). **p** Fmoc-F-F (Gly). **q** Fmoc-F-F (Phe). **r** Fmoc-F-F (Ile). **s** Fmoc-F-F (Met). Three times each experiment was repeated independently with similar results. Representative images are shown.

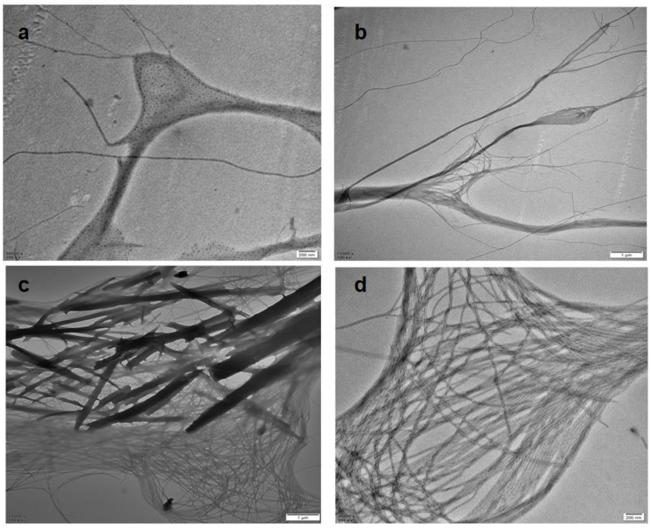

Supplementary Fig. 3 Fmoc-F-F (His) co-assembly at different sonication time. **a** and **b** 10 min. **c** 20 min. **d** 45 min. Three times each experiment was repeated independently with similar results. Representative images are shown.

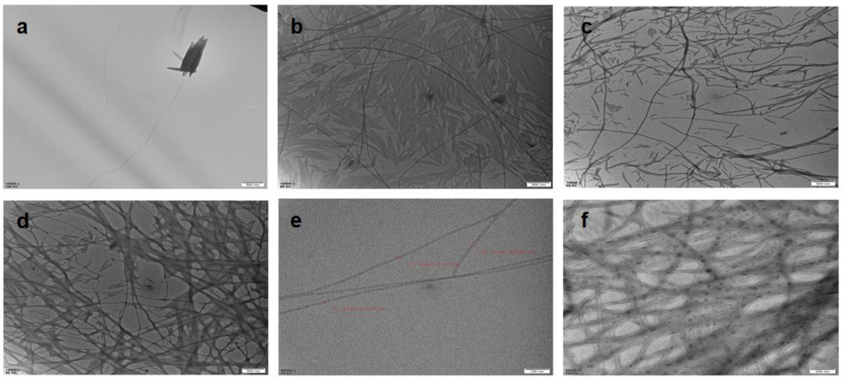

Supplementary Fig. 4 TEM characterization for Fmoc-F-F (His) co-assembly with different His concentrations (Fmoc-F-F was fixed at 2mg mL^{-1}). **a** 2 mg mL^{-1}. **b** 10 mg mL^{-1}. **c** 12 mg mL^{-1}. **d** 14 mg mL^{-1}. **e** 18 mg mL^{-1}. **f** 30 mg mL^{-1}. Three times each experiment was repeated independently with similar results. Representative images are shown.

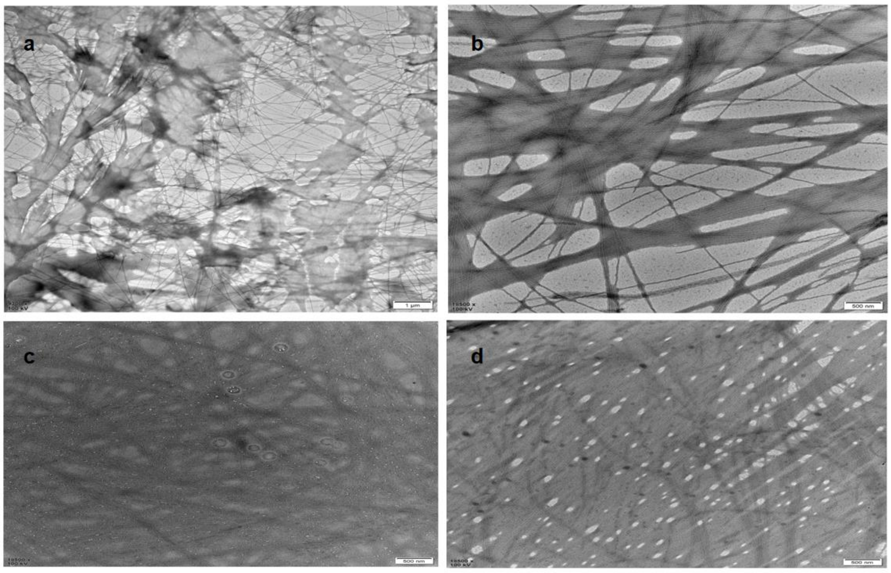

Supplementary Fig. 5 TEM characterization for Fmoc-F-F (His) co-assembly with different Fmoc-F-F concentrations (His was fixed at 20 mg mL^{-1}). **a** 0.5 mg mL^{-1}. **b** 1 mg mL^{-1}. **c** 3 mg mL^{-1}. **d** 10 mg mL^{-1}. Three times each experiment was repeated independently with similar results. Representative images are shown.

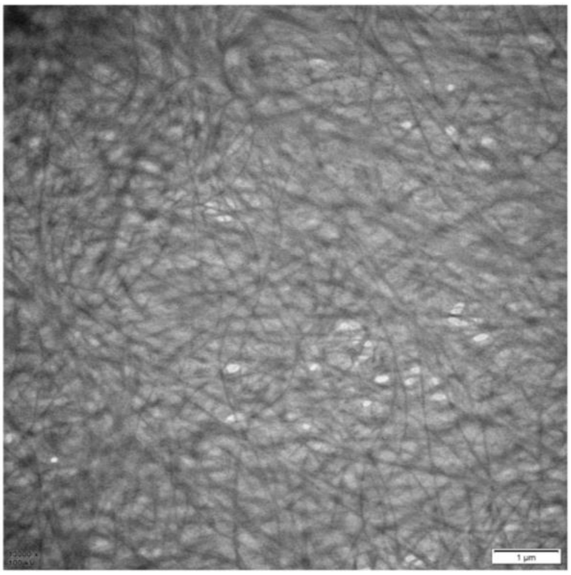

Supplementary Fig. 6 Fmoc-F-F (His) filaments formed in low-resistance water. Three times each experiment was repeated independently with similar results. Representative image is shown.

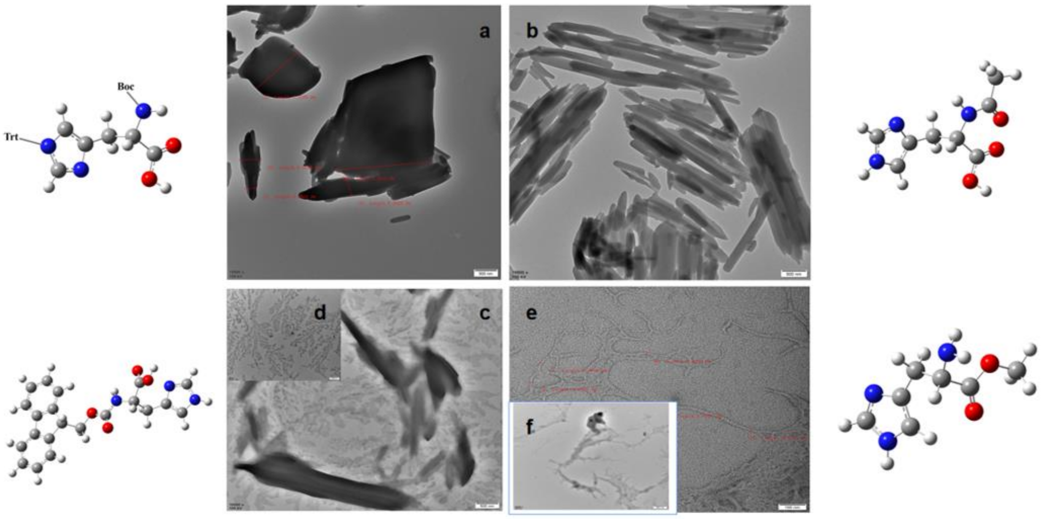

Supplementary Fig. 7 TEM characterization for Fmoc-F-F (His) co-assembly with side-chain modified His. **a** N-Boc-N'-trityl-L-histidine (Boc-His(Trt)-OH). **b** N-Acetyl-L-histidine (N-Acetyl-L-His). **c** and **d** Fmoc-His. **e** and **f** L-Histidine Methyl Ester Dihydrochloride (His-OMe). Three times each experiment was repeated independently with similar results. Representative images are shown.

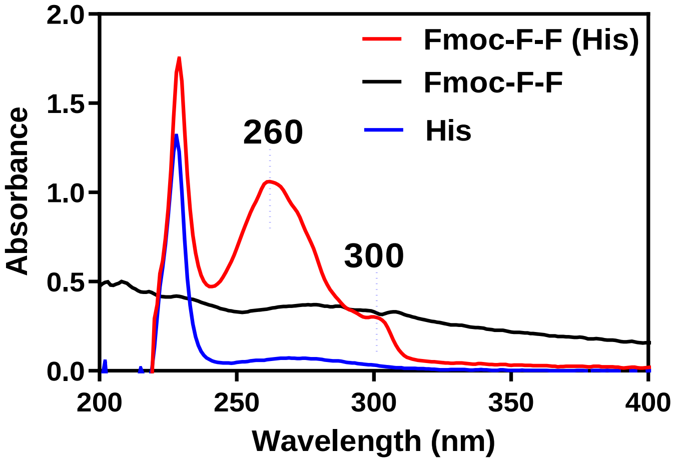

Supplementary Fig. 8 UV-VIS characterization of functional group of Fmoc-F-F(His). UV-VIS assay confirmed the presence of benzene ring and Fmoc in Fmoc-F-F (His) nanofilaments, indicating the interaction of His and Fmoc-F-F.

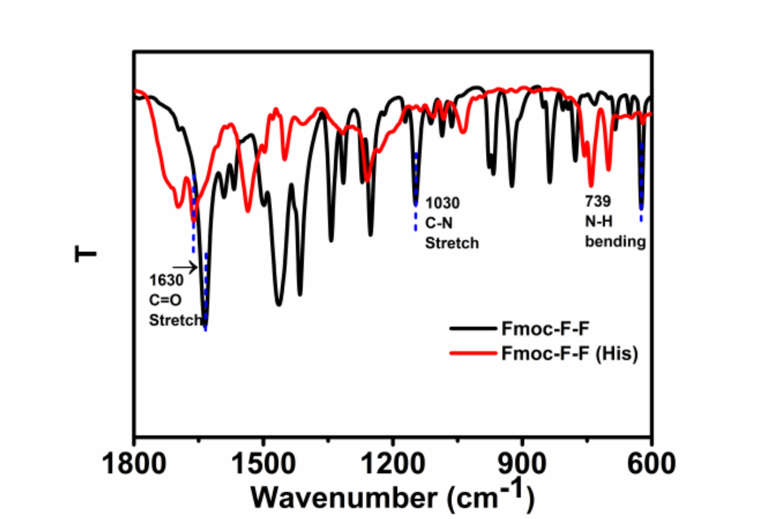

Supplementary Fig. 9 FTIR characterization of non-covalent interactions between His and Fmoc-F-F. FTIR spectra (600-1800 cm^{-1}) of Fmoc-F-F (His) and Fmoc-F-F were provided. C-N stretching (1 030 cm^{-1}), N-H bending (739 cm^{-1}, 3 300 cm^{-1}) and C=O (1 630 cm^{-1}) of Fmoc-F-F (His) were compared to Fmoc-F-F.

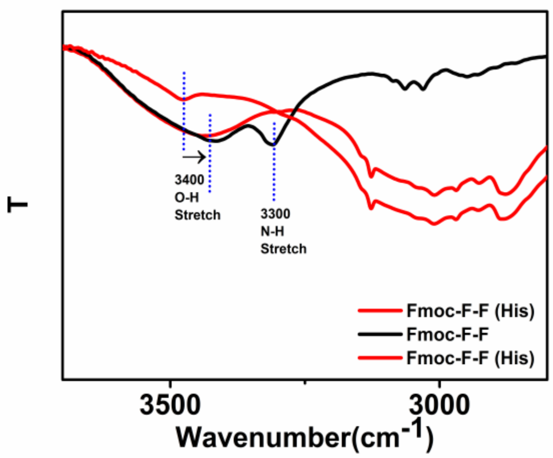

Supplementary Fig. 10 FTIR characterization of non-covalent interactions between His and Fmoc-F-F. FTIR spectra (2 800-3 700 cm^{-1}) were provided. -OH stretch (3 400 cm^{-1}) and N-H stretch (3 300 cm^{-1}) of Fmoc-F-F (His) were compared to Fmoc-F-F.

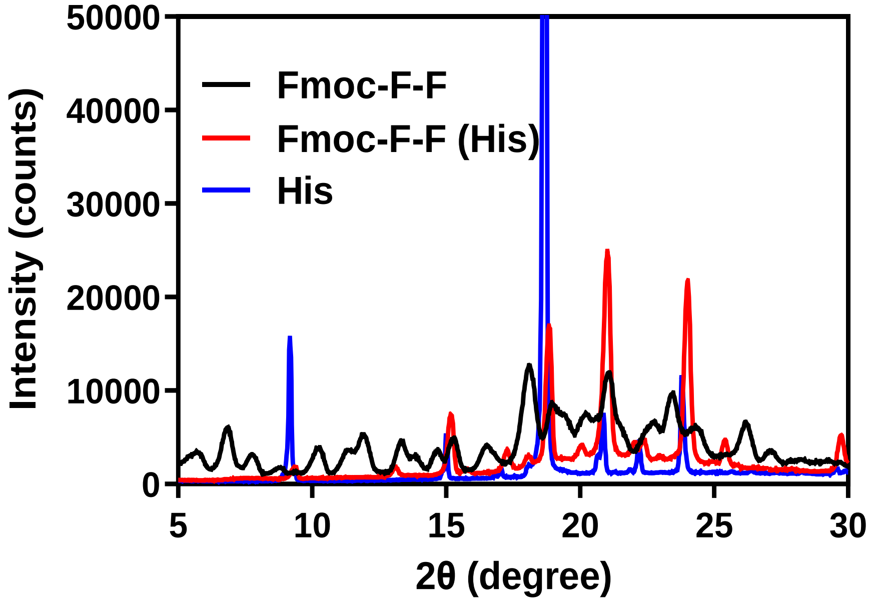

Supplementary Fig. 11 X-ray diffraction (XRD) characterization of Fmoc-F-F (His). The XRD was used to confirm the interaction between Fmoc-F-F and His in Fmoc-F-F (His) nanofilaments. Compared to Fmoc-F-F or His, strong diffraction peaks at 21° and 24° were formed in Fmoc-F-F (His).

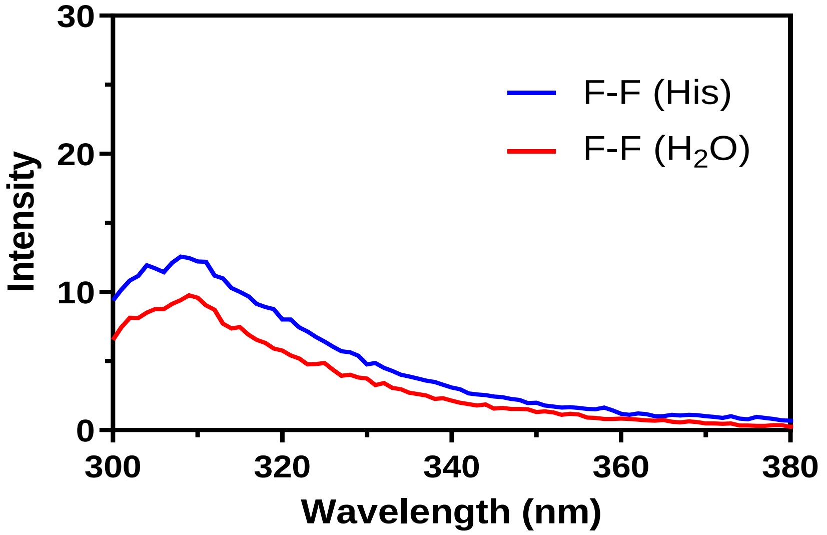

Supplementary Fig. 12 Fluorescence emission spectrum of F-F (His) and F-F. In the absence of Fmoc, both F-F and F-F (His) showed weak fluorescence intensity between 300-320 nm

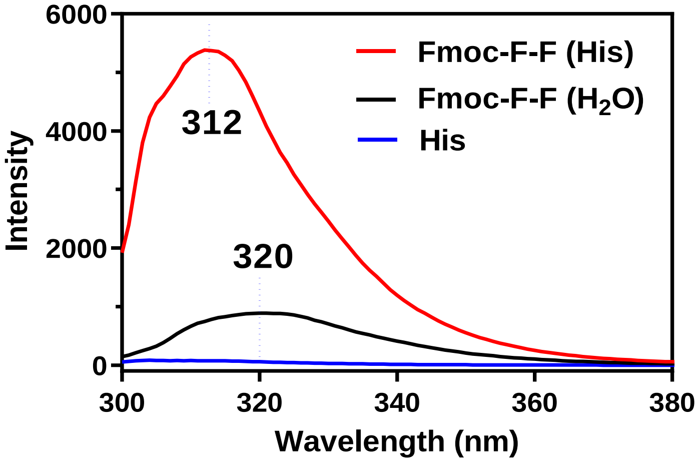

Supplementary Fig. 13 Fluorescence emission spectrum of Fmoc-F-F (His). The enhanced intensity of Fmoc-F-F (His) between 300-320 nm confirmed the strong interaction between Fmoc-F-F and His, and the characteristic peak was shifted from 320 nm to 312 nm, indicating that the π-π action of Fmoc-F-F was weakened in the presence of His.

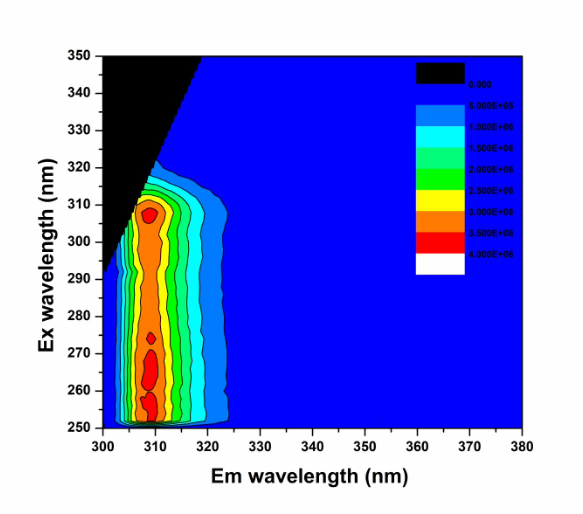

Supplementary Fig. 14 Three-dimensional fluorescence spectrum of Fmoc-F-F (His) nanofilaments. Three-dimensional fluorescence spectrum of Fmoc-F-F (His) nanofilaments were collected when setting the Em wavelength and Ex wavelength from 300-380 nm, 250-350 nm, respectively. The strong intensity were demonstrated at ~310 nm, which is consistent with the results of Supplementary Fig. 13.

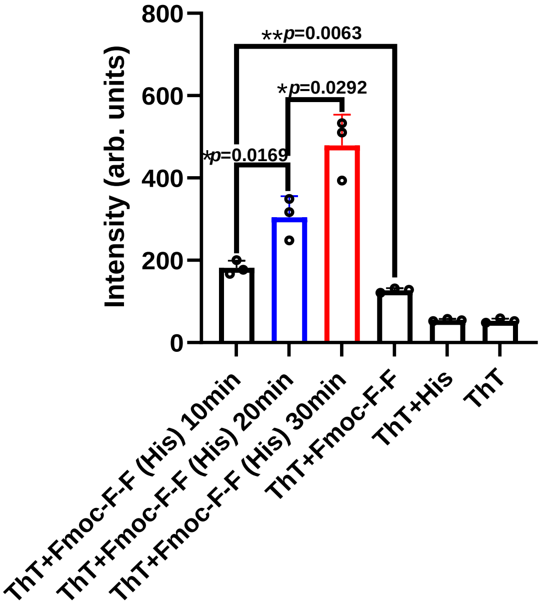

Supplementary Fig. 15 ThT-binding assay for the formation of amyloid-like filaments by Fmoc-F-F (His). n=3 independent samples, bars represent means ± SD. The significant difference was evaluated by two-tailed unpaired t-test.***$p < 0.01$, *$p < 0.05$.

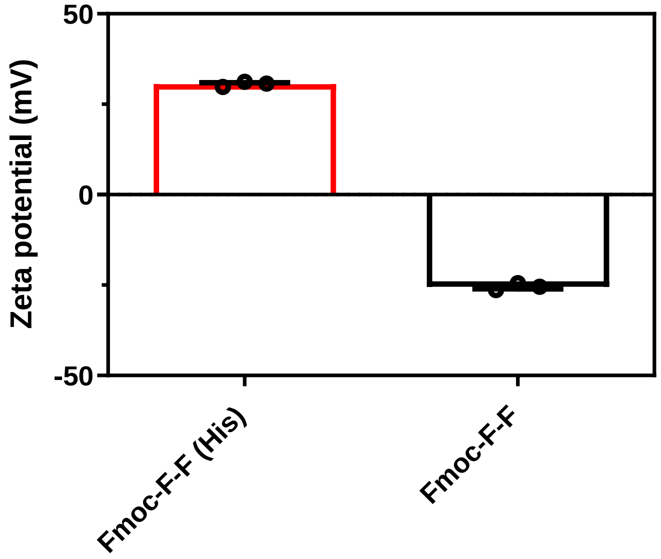

Supplementary Fig. 16 Zeta potential assay for the electrostatic interaction between His and Fmoc-F-F. The Fmoc-F-F (His) showed the positive potentials, while Fmoc-F-F was negative charged, indicating that His led to the remarkable charge inversion when adding His into Fmoc-F-F system. n=3 independent samples, bars represent means ± SD. The significant difference was evaluated by two-tailed unpaired t-test. $^{**}p < 0.01$, $^{*}p < 0.05$.

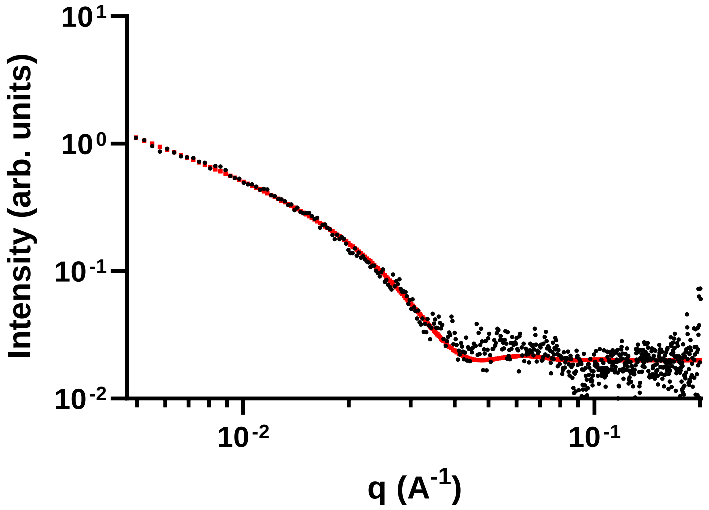

Supplementary Fig. 17 The SAXS data of Fmoc-F-F (His) nanofilaments. The fitting was conducted using SasView software and the radius (R) of nanofilaments was estimated at 8.18 nm.

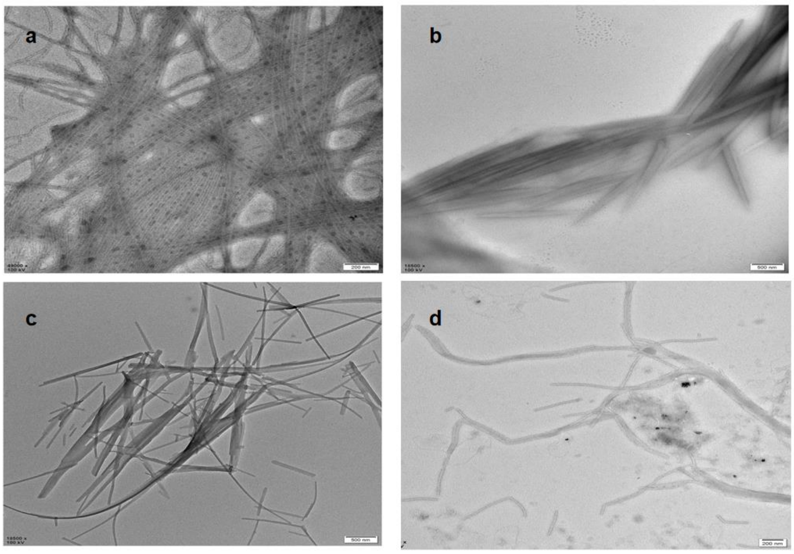

Supplementary Fig. 18 Dynamic balance of Fmoc-F-F (His). a His changed the morphology of pre-assembled Fmoc-F-F nanorods . When His was added to pre-assembled Fmoc-F-F nanorods, filaments could also appear, but the degree of fibrillogenesis was obviously weaker than simultaneous co-assembly of Fmoc-F-F and His, indicating that His could depolymerize self-assembled Fmoc-F-F nanorods into nanofilaments. **b** The morphology of co-assembled Fmoc-F-F (His) at low concentration of Fmoc-F-F (0.2 mg mL^{-1}) and His (2 mg mL^{-1}). Low concentrations of Fmoc-F-F and His formed shorter filaments, indicating that the growth of filaments is dependent on the concentration of co-assembly moieties. **c** The morphology of Fmoc-F-F (His) nanofilaments after removing His by dialysis. The 3 kD molecular weight dialysis membrane was used to to remove free His. After dialysis, filaments had a tendency to aggregate into nanorods, indicating that even free His plays an important role in maintaining the degree of fibrillogenesis of Fmoc-F-F (His). **d** The morphology of Fmoc-F-F (His) nanofilaments after removing His using centrifugation. After centrifuging Fmoc-F-F (His) at 3600 xg for 5 min, filaments became shorter, further demonstrating that His play a critical role in the assembly of nanofilaments. Three times each experiment was repeated independently with similar results. Representative images are shown.

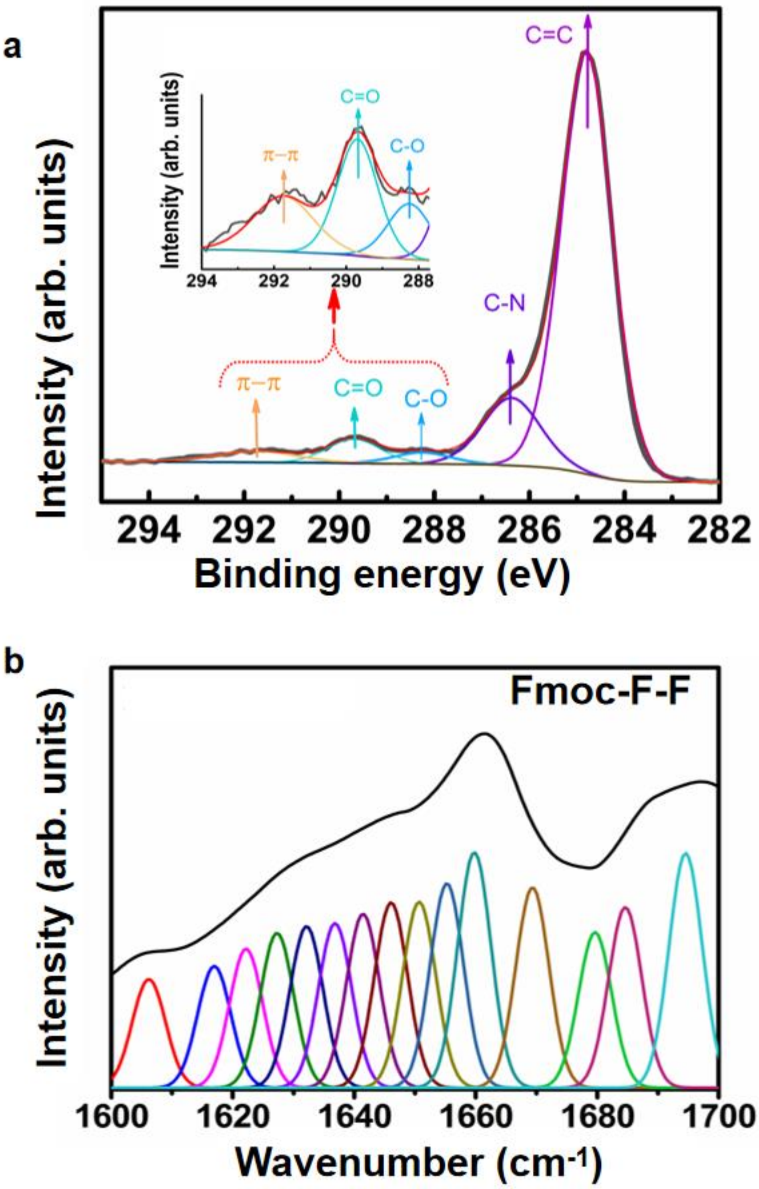

Supplementary Fig. 19 The characterization of π-π stacking and secondary structure of Fmoc-F-F. **a** C *1s* peaks of Fmoc-F-F nanofilaments analyzed by X-ray photoelectron spectroscopy (XPS). **b** Percentage of secondary structure for the amide I region (1 600 cm^{-1} to 1 700 cm^{-1}) of Fmoc-F-F nanofilaments characterized by FTIR.

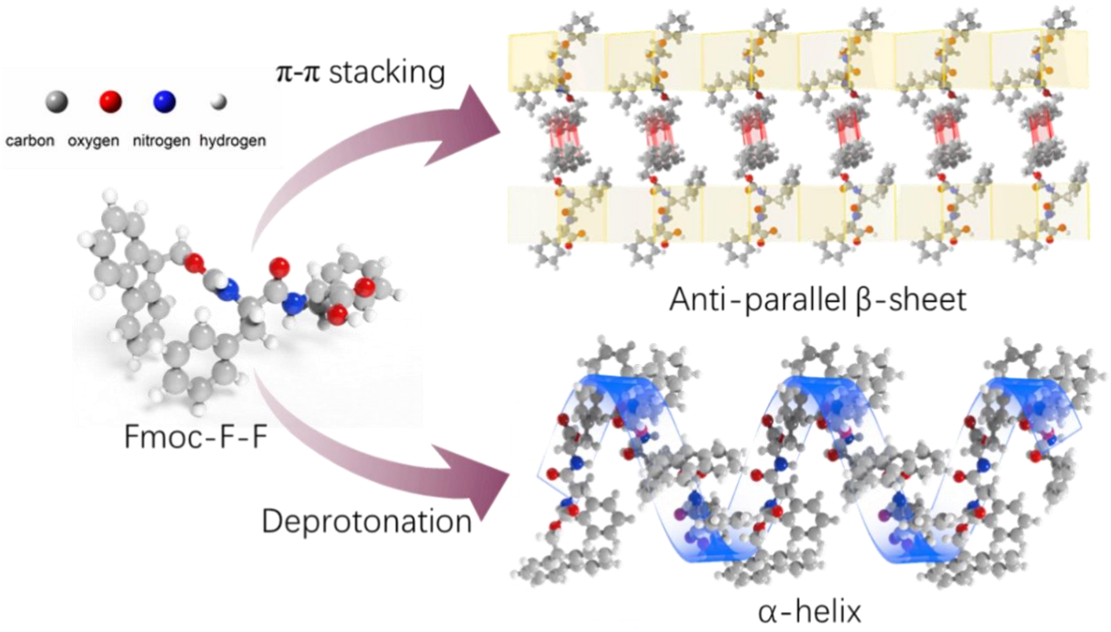

Supplementary Fig. 20 Proposed mechanism for the self-assembly of Fmoc-F-F driven by anti-parallel β-sheet and a-helix secondary structures. Two main secondary structures were formed in the assembly of Fmoc-F-F owing to the π-π stacking and deprotonation, respectively.

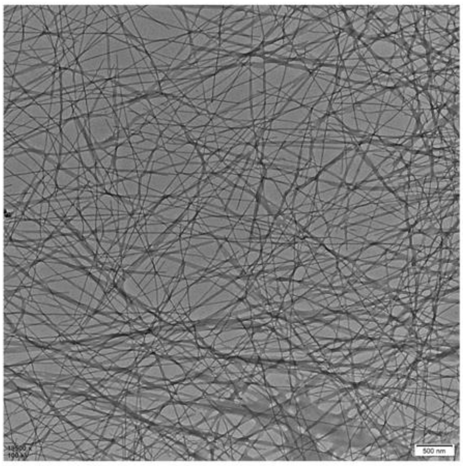

Supplementary Fig. 21 TEM characterization for Fmoc-F-F (His) nanofilaments. The as-prepared Fmoc-F-F (His) nanofilaments were stored at room temperature for 60 days. 3 times each experiment was repeated independently with similar results. Representative image is shown.

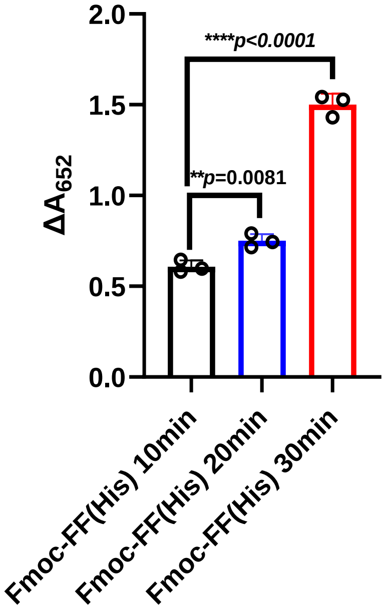

Supplementary Fig. 22 POD-activity of Fmoc-F-F (His) assembled under different assembly time. n=3 independent samples, bars represent means ± SD, The significant difference was evaluated by two-tailed unpaired t-test. ****p < 0.0001, **p < 0.01.

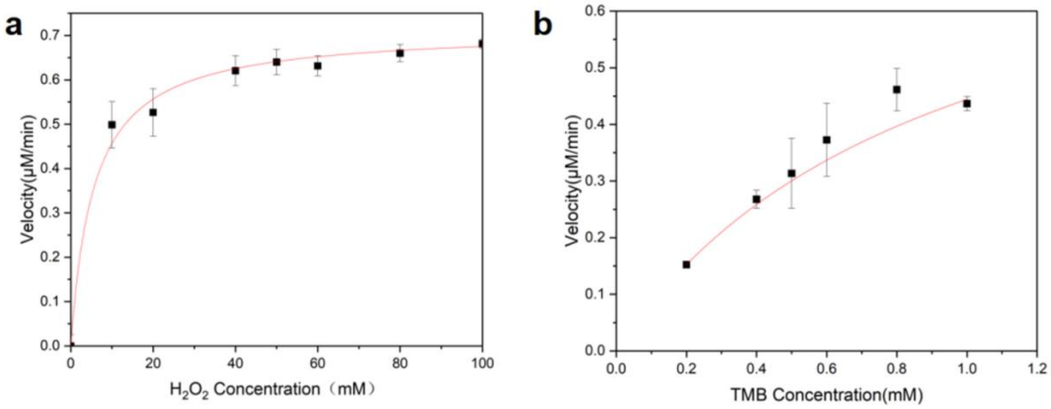

Supplementary Fig. 23 Enzymatic kinetics assays for Fmoc-F-F (His) nanofilaments. **a** Michaelis-Menten curves regarding to variable H_2O_2 concentrations. The concentration of TMB was fixed at 1.0 mM; b Michaelis-Menten curves regarding to variable TMB concentrations. The concentration of H_2O_2 was fixed at 10 mM. n=3 independent samples, bars represent means ± SD.

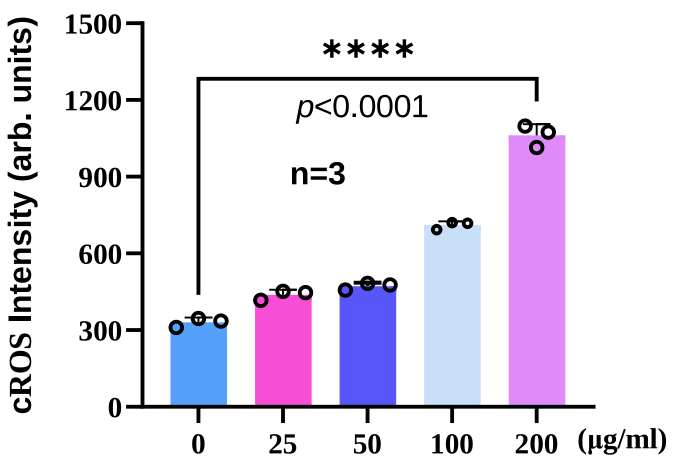

Supplementary Fig. 24 Cytosolic ROS (cROS) level in PC12 cells treated with Fmoc-F-F (His) nanofilaments. The significant difference was evaluated by two-tailed unpaired t-test. ****p < 0.0001.

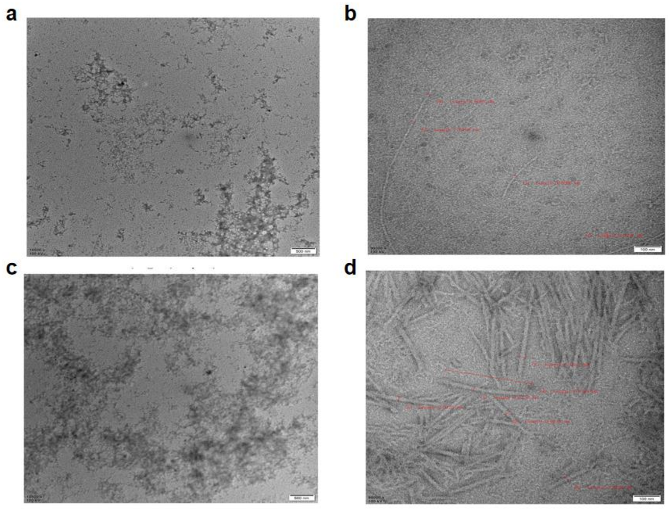

Supplementary Fig. 25 TEM characterization for the morphology of Aβ1-40 and Aβ1-42 assemblies. **a** Aβ1-40 Oligomer. **b** Aβ1-40 incubated at 37℃ for 14days. **c** Aβ1-42 Oligomer. **d** Aβ1-42 incubated at 37°C for 14days. Three times each experiment was repeated independently with similar results. Representative images are shown.

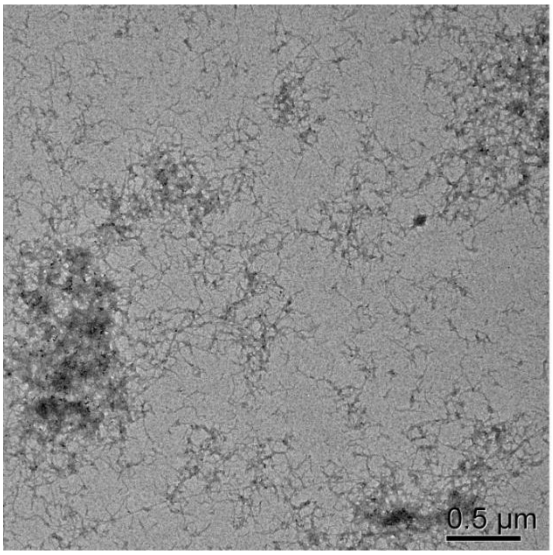

Supplementary Fig. 26 TEM characterization for Aβ1-40 aggregates incubated at 37℃ for half a year. Three times each experiment was repeated independently with similar results. Representative image is shown.

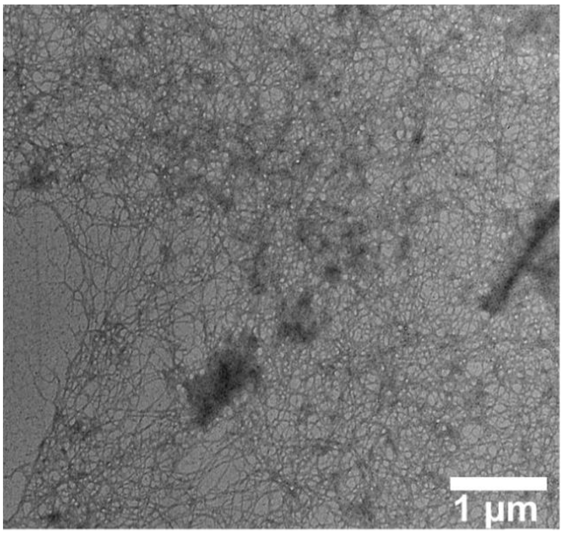

Supplementary Fig. 27 TEM characterization for Aβ1-42 aggregates incubated at 37℃ for half a year. Three times each experiment was repeated independently with similar results. Representative image is shown.

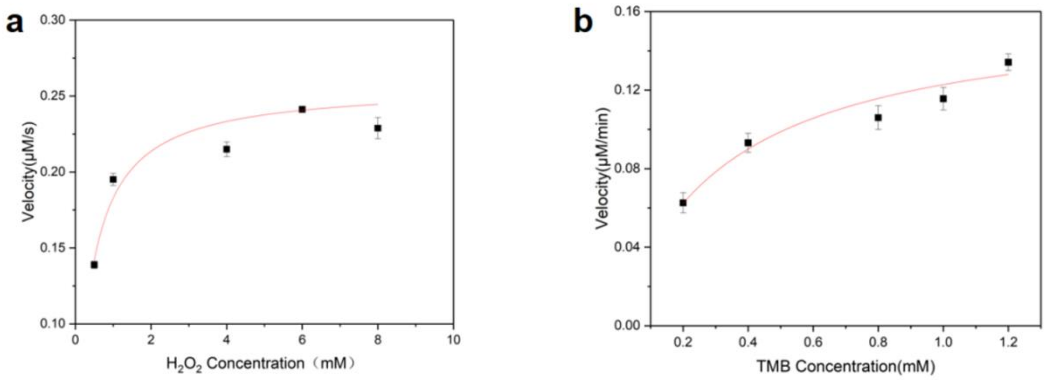

Supplementary Fig. 28 Enzymatic kinetics assays of Aβ filament. **a** Michaelis-Menten curves regarding to variable H_2O_2 concentrations. The concentration of TMB was fixed at 1.0 mM; **b** Michaelis-Menten curves regarding to variable TMB concentrations. The concentration of H_2O_2 was fixed at 10 mM. n=3 independent samples, bars represent means ± SD.

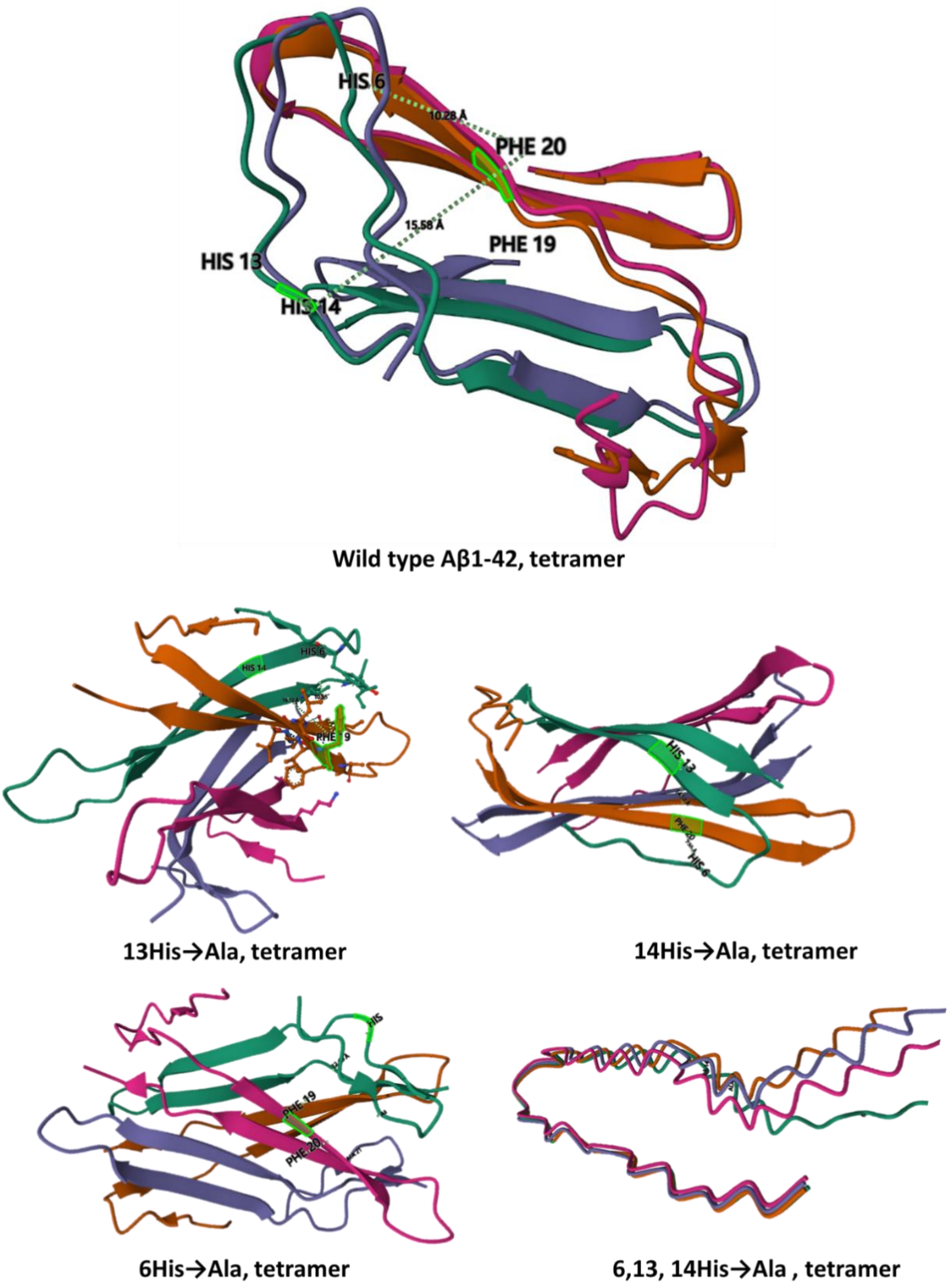

Supplementary Fig. 29 Alphafold2 predicted assembled tetramer structure of wild type Aβ1-42 and different His mutants. The distance between His and F-F (19-20) are marked. There are three His residues in Aβ1-42 (position 6, 13 and 14). To analyze the role of each His in Aβ1-42 assembly, alanine (Ala) was used to replace His one by one when using Alphafold2 for the prediction.

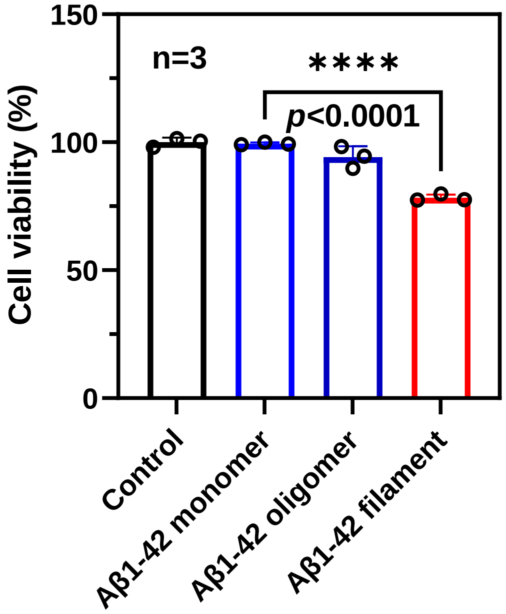

Supplementary Fig. 30 The cytotoxicity of different assemblies of Aβ 1-42 toward HT-22 cells. n=3 independent cells, bars represent means ± SD. The significant difference was evaluated by two-tailed unpaired t-test. ****$p < 0.0001$.

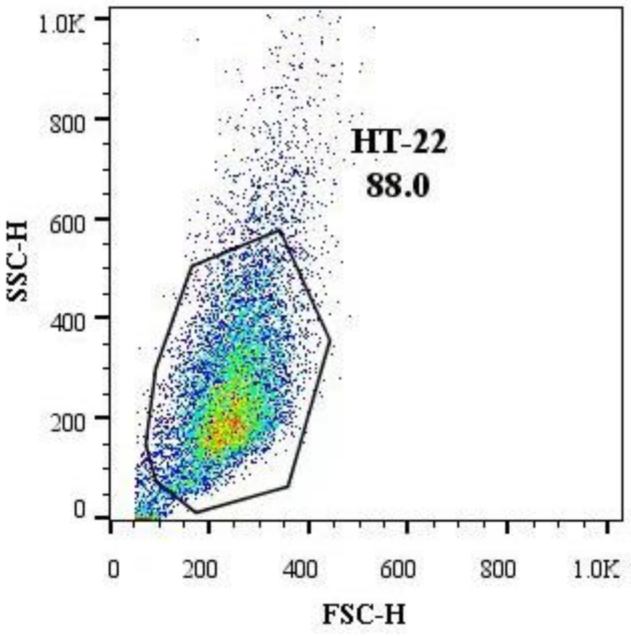

Supplementary Fig. 31 The representative figure for exemplifying the gating strategy of HT-22 corresponding to ROS of mitochondria (Supplementary Fig. 32). Similar gating strategies were conducted in Fig. 5g and h, Supplementary Fig. 24.

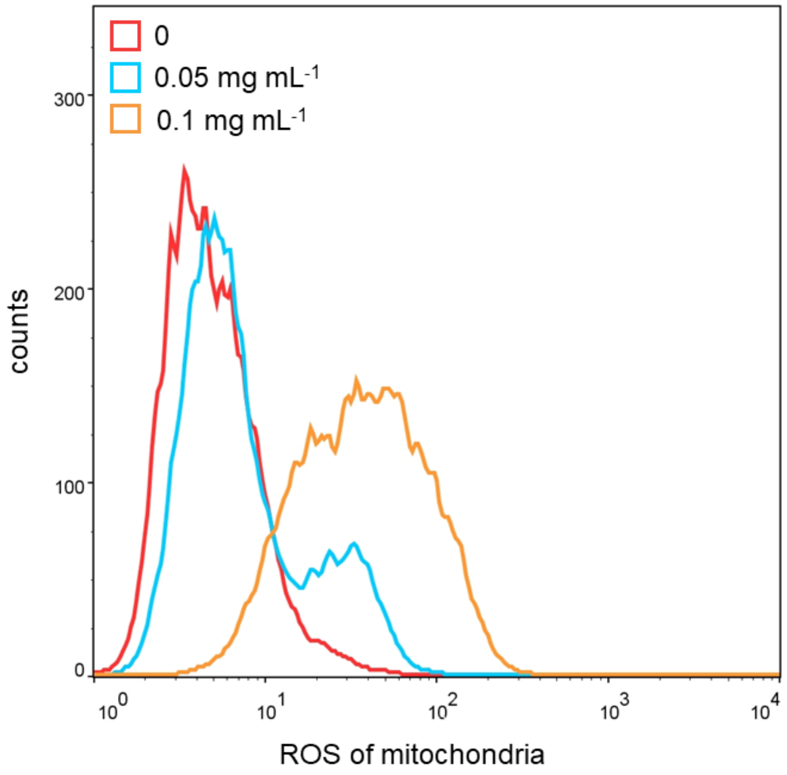

Supplementary Fig. 32 The assessment of cellular ROS of mitochondria. HT-22 cells were treated by different concentrations of Aβ1-42 filaments and the fluorescence intensity of ROS of mitochondria were collected by flow cytometry.

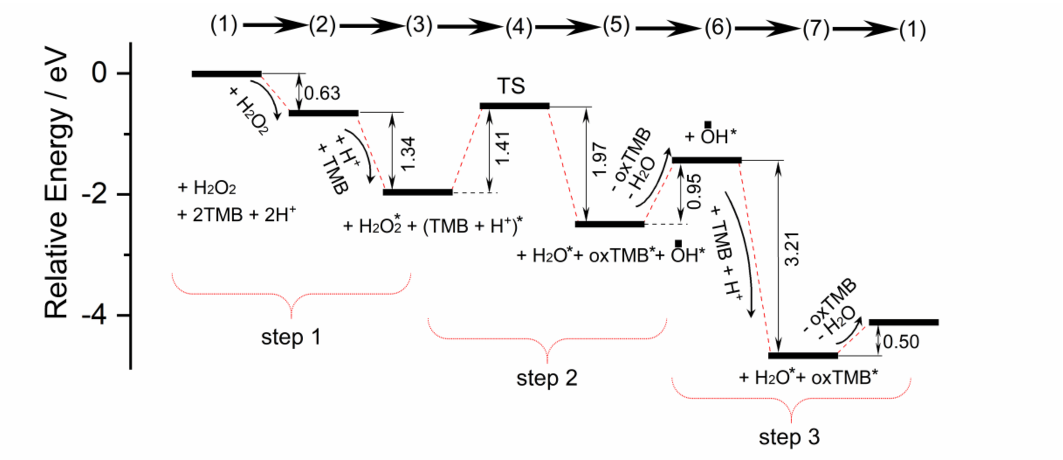

Supplementary Fig. 33 Relative energies for key intermediate in the peroxidase catalytic cycle. Energy unit: eV. Three steps were proposed: (1) adsorption of H_2O_2 (0.63eV) and (H^+ + TMB) molecules on Fmoc-F-F (His) (1.34eV) (states 1 to states 3); (2) oxidation reaction of the first TMB molecule oxidized by H_2O_2* under acidic condition (1.41eV), producing HO*, H_2O*, and oxTMB* (1.97eV); and (3) oxidation of the second TMB molecule by second HO* under acidic condition (0.95eV), producing H_2O* and oxTMB* (3.21eV). The corresponding states were shown in Fig. 6.

Supplementary Table 1 The co-assembly properties of amino acid & Fmoc-F-F.

Amino acid	Side chain	Co-assembly morphology	classification of Amino acid
Asn	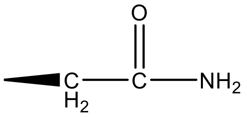	Nanofiber, nanorod	Polarity uncharged
Asp	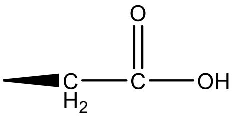	Nanofiber	Negative charge
Ser	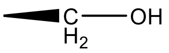	Nanofiber, nanorod	Polarity uncharged
Lys	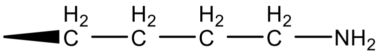	Nanofiber	positive charge
Arg	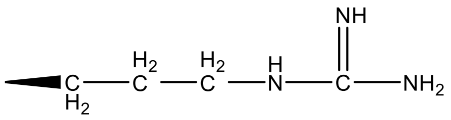	Nanofiber	positive charge

Pro	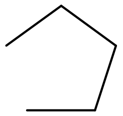	Nanofiber, nanorod	Non-polar
Leu	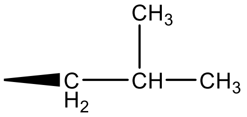	Nanofiber	Non-polar
Tyr	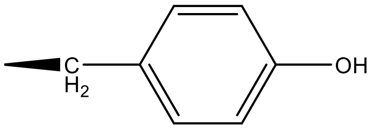	Spherical arrangement of nanorod	Polarity uncharged
Thr	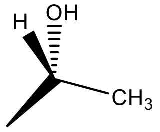	Spherical arrangement of nanorod	Polarity uncharged
Cys	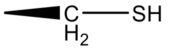	Spherical arrangement of nanorod	Polarity uncharged
Ala	$-CH_3$	nanorod	Non-polar
Glu	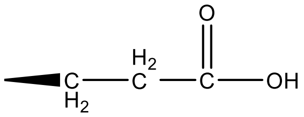	nanorod	Negative charge
Val	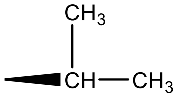	nanorod	Non-polar
Trp	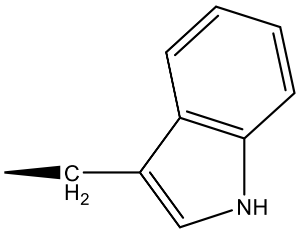	nanorod	Non-polar
Gln	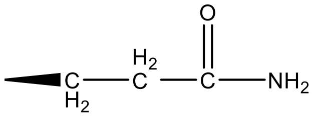	nanorod	Polarity uncharged
Gly	$-H$	nanorod	
Phe	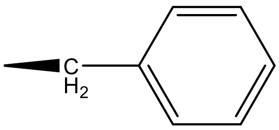	nanorod	Non-polar
Ile	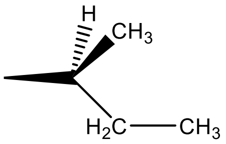	nanorod	Non-polar
Met	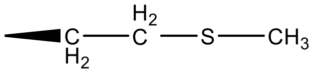	nanorod	Non-polar

Supplementary Table 2 The percent of different secondary structures in the Fmoc-F-F (His) filaments.

Wavenumber	Structure	Percent
1610-1640 cm^{-1}	β-Sheet	71.60%
1640-1650 cm^{-1}	Radom coils	21.90%
1650-1660 cm^{-1}	α-helix	3.10%
1660-1695 cm^{-1}	Antiparallel β-sheet	3.40%

Supplementary Table 3 The percent of different secondary structures in the Fmoc-F-F nanorods.

Wavenumber	Structure	Percent
1610-1640 cm^{-1}	β-Sheet	26.30%
1640-1650 cm^{-1}	Radom coils	11.20%
1650-1660 cm^{-1}	α-helix	19.50%
1660-1695 cm^{-1}	Antiparallel β-sheet	43.20%
