# Supplementary material for: Histidine modulates amyloid-like assembly of peptide nanomaterials and confers enzyme-like activity
Source: Nat Commun. 2023 Sep 19;14:5808. doi: 10.1038/s41467-023-41591-1 (PMC10509148; doi:10.1038/s41467-023-41591-1)
Supplement: Supplementary file 3 — Description of Additional Supplementary Files [file 41467_2023_41591_MOESM3_ESM.pdf]

<u>Description of Additional Supplementary Files</u>

Supplementary Data 1: The optimized atomic coordinates of the Fmoc-F-F molecule.
