# Supplementary material for: Histidine modulates amyloid-like assembly of peptide nanomaterials and confers enzyme-like activity
Source: Nat Commun. 2023 Sep 19;14:5808. doi: 10.1038/s41467-023-41591-1 (PMC10509148; doi:10.1038/s41467-023-41591-1)
Supplement: Supplementary file 4 — Supplementary Data 1 [file 41467_2023_41591_MOESM4_ESM.pdf]

Supplementary Data 1. The optimized atomic coordinates of the Fmoc-F-F molecule.

H	-4.36164705	-2.69779039	0.71974451
C	-4.53431549	1.83646845	2.42016564
C	-3.35229200	1.09481717	2.39061476
C	-3.28363954	-0.10783142	1.68574401
C	-4.40824248	-0.55083639	1.00371282
C	-5.60284198	0.18859974	1.04628963
C	-5.67077405	1.38631610	1.75300101
H	-4.56966795	2.77066023	2.97422696
H	-2.47668380	1.45512085	2.92430999
H	-2.36148330	-0.68175735	1.67140353
H	-6.59322567	1.96054729	1.78684789
C	-4.58868733	-1.78592898	0.15040048
C	-6.05040692	-1.70963676	-0.22614158
C	-6.80623952	-2.59058867	-0.98510052
C	-8.14685277	-2.28965756	-1.23284876
C	-8.71564174	-1.11952064	-0.72804013
C	-7.95928944	-0.23135520	0.03339551
C	-6.62276144	-0.53078184	0.28224984
H	-6.36904650	-3.50587141	-1.37780824
H	-8.75272965	-2.97265393	-1.82203312
H	-9.76070648	-0.90007004	-0.92990525
H	-8.40771747	0.67787338	0.42566572
C	-3.71848236	-1.78057823	-1.10981697
H	-3.92716267	-2.66747123	-1.71587641
H	-3.90396020	-0.88246491	-1.70392855
O	-2.33979346	-1.84205480	-0.75476926
C	-1.61742677	-0.71308133	-0.95736929
O	-2.00781123	0.27444541	-1.55232062
N	-0.39980461	-0.83166372	-0.38597657
H	-0.07545639	-1.70601946	0.00768476
C	0.55043902	0.24007405	-0.48354464
H	0.57855280	0.60463103	-1.51895384
C	0.16644382	1.43531602	0.43018488
H	-0.92768374	1.47354030	0.43685711
H	0.49529216	1.23326664	1.45661624
C	0.70165640	2.74925555	-0.06961823
C	0.14054407	3.31554778	-1.22168221
C	1.76385101	3.40596298	0.55401496
C	0.63465545	4.50919660	-1.73406911
H	-0.69089846	2.80537385	-1.70508714

C	2.26546250	4.59897903	0.03662828
H	2.21286116	2.97340634	1.44530520
C	1.70118245	5.15336708	-1.10704312
H	0.18685868	4.93977290	-2.62595277
H	3.10465037	5.08560189	0.52525994
H	2.09113089	6.08332815	-1.51204306
C	1.91226810	-0.34643606	-0.12056673
O	2.02349077	-1.49219515	0.31020713
N	2.97439392	0.46771693	-0.28686319
H	2.86807304	1.43428017	-0.57602281
C	4.28801435	0.01602231	0.09901031
H	4.21236692	-0.52258575	1.05061446
C	5.16319533	1.23207307	0.29400405
O	4.87418467	2.35269295	-0.05906705
O	6.32272529	0.91870668	0.88124898
H	6.83956699	1.74138323	0.93111818
C	4.89841744	-0.95596222	-0.94660225
H	4.05677582	-1.54689655	-1.32087702
H	5.29615688	-0.38289143	-1.79225714
C	5.93490176	-1.88082659	-0.37046188
C	7.29071542	-1.74932893	-0.66884876
C	5.52863191	-2.90073107	0.49625361
C	8.22656115	-2.61912638	-0.11493936
H	7.61605073	-0.95708229	-1.33954686
C	6.46155177	-3.76743235	1.05470278
H	4.46927554	-3.00770665	0.72409059
C	7.81445066	-3.62902584	0.74970297
H	9.27970590	-2.50694862	-0.35939906
H	6.13199425	-4.55725528	1.72469735
H	8.54397145	-4.30843080	1.18244168

The optimized atomic coordinates of the Dimer 1

H	6.45874014	2.38753700	0.29694367
C	5.64414744	-1.70768855	2.75797563
C	4.70341026	-0.67934482	2.68241947
C	4.87098983	0.37431058	1.78235346
C	5.98486472	0.37826396	0.95456119
C	6.94116516	-0.64778094	1.04331407
C	6.77371893	-1.69682804	1.94348173
H	5.49788877	-2.51997199	3.46489490
H	3.83380123	-0.69569458	3.33433475

H	4.13664779	1.17313341	1.73190969
H	7.51132179	-2.49234163	2.01374297
C	6.38183910	1.37159240	-0.11378687
C	7.72322368	0.84216146	-0.56710400
C	8.59190978	1.35598007	-1.51829196
C	9.76893582	0.66098117	-1.80282720
C	10.06434415	-0.53273723	-1.14322992
C	9.19370607	-1.05265851	-0.18800876
C	8.02021727	-0.36048638	0.09737946
H	8.36857597	2.28795923	-2.03271057
H	10.46154190	1.05381419	-2.54217075
H	10.98498514	-1.06113581	-1.37606300
H	9.42983126	-1.98185628	0.32432370
C	5.40858286	1.40125452	-1.29650128
H	5.75026150	2.11718327	-2.04983079
H	5.32325182	0.41004028	-1.74788335
O	4.12133808	1.84173320	-0.86907484
C	3.15417830	0.89694377	-0.81978244
O	3.25658510	-0.23641313	-1.25407446
N	2.05719383	1.37806391	-0.19211757
H	1.93217241	2.37299672	-0.04514955
C	0.87780787	0.56207732	-0.11052175
H	0.68901205	0.10164675	-1.09190548
C	1.03441883	-0.58305760	0.92543310
H	2.10764668	-0.78373425	1.00291306
H	0.69681402	-0.24256847	1.91141647
C	0.33921165	-1.86260628	0.53308464
C	0.83399771	-2.60297355	-0.54974076
C	-0.77290558	-2.34685854	1.22672097
C	0.24372918	-3.81288271	-0.90552527
H	1.70472847	-2.22825888	-1.08467699
C	-1.38017299	-3.54644526	0.85666480
H	-1.17497195	-1.77903768	2.06239405
C	-0.86356652	-4.29022822	-0.19948869
H	0.66063169	-4.39474966	-1.72439919
H	-2.26003718	-3.89269981	1.39112835
H	-1.32202016	-5.23643934	-0.47593092
C	-0.28966638	1.50410588	0.19256233
O	-0.14867301	2.72299347	0.20945891
N	-1.47301719	0.89754066	0.40311524
H	-1.53042447	-0.10930970	0.50024187

C	-2.67728872	1.65574674	0.65442401
H	-2.45279585	2.43831060	1.39236857
C	-3.68261611	0.70109824	1.26755968
O	-3.33827044	-0.39095432	1.71134634
O	-4.90924295	1.14573032	1.25718550
H	-5.53492520	0.39001309	1.45552875
C	-3.22043609	2.33586205	-0.62591397
H	-2.34341369	2.57890713	-1.23503801
H	-3.83355062	1.61853229	-1.18239478
C	-3.98231575	3.60383021	-0.34767779
C	-5.36334223	3.68371981	-0.52562617
C	-3.29011054	4.73465152	0.09807303
C	-6.04239978	4.87109215	-0.26544203
H	-5.90650294	2.80398724	-0.86078711
C	-3.96737363	5.92017645	0.36352951
H	-2.21061567	4.67452476	0.22906400
C	-5.34710760	5.99165481	0.18105702
H	-7.11873620	4.91975143	-0.40946270
H	-3.41685849	6.79218637	0.70736356
H	-5.87763420	6.91808282	0.38510308
N	-8.14511453	-2.95679918	-0.03779905
H	-8.47444439	-1.99493500	0.02521817
C	-6.70182343	-2.93024658	-0.16194675
H	-6.30577252	-3.94576492	-0.05655180
C	-6.32836574	-2.38869429	-1.56316562
C	-6.00643070	-2.04086985	0.84877913
H	-6.75221422	-3.08518114	-2.29356655
H	-6.83375975	-1.42481445	-1.69389967
C	-4.86524097	-2.17473070	-1.77793734
O	-6.48633774	-0.96682666	1.19669273
N	-4.29826391	-0.92730243	-1.63173129
C	-3.88790780	-3.09838425	-2.05743937
C	-3.00998243	-1.10088100	-1.82749344
N	-2.71382088	-2.39324470	-2.09587774
H	-3.92277881	-4.16481658	-2.22355688
H	-2.25600316	-0.32591725	-1.80262204
H	-1.78440372	-2.78617018	-2.15924353
O	-4.83871317	-2.49957209	1.24753113
H	-8.40723867	-3.39800967	0.84012785
H	-4.32315371	-1.75125195	1.65564559

The optimized atomic coordinates of the Dimer 2

H	4.18075956	1.30749344	-1.54079386
C	4.17529025	-0.21226804	3.03470882
C	2.98916788	-0.00769818	2.32860627
C	3.01088055	0.17221604	0.94430222
C	4.22897391	0.14696053	0.27836590
C	5.42306412	-0.05748977	0.99103218
C	5.40042110	-0.23953580	2.37165133
H	4.14357114	-0.34929921	4.11221620
H	2.03806319	0.01785564	2.85395872
H	2.08729689	0.31948579	0.39662542
H	6.32221866	-0.39625794	2.92609798
C	4.52027365	0.32036289	-1.19485082
C	6.02675720	0.21420671	-1.24174141
C	6.87624332	0.30141138	-2.33449449
C	8.24951262	0.14985857	-2.13505273
C	8.75921430	-0.08467027	-0.85718367
C	7.90954064	-0.17153682	0.24322087
C	6.53969013	-0.02247037	0.04554298
H	6.48512651	0.48984271	-3.33166135
H	8.92762152	0.21738118	-2.98133786
H	9.83106790	-0.19899124	-0.71930406
H	8.31186129	-0.35258547	1.23671182
C	3.85525185	-0.73570139	-2.07875989
H	4.14596075	-0.59387675	-3.12455512
H	4.12925243	-1.74603392	-1.76511321
O	2.44207206	-0.57386617	-2.00189520
C	1.74041506	-1.61032846	-1.48725167
O	2.16902007	-2.73638468	-1.32223675
N	0.47938691	-1.20907955	-1.18628449
H	0.26974661	-0.20835850	-1.19268617
C	-0.37278930	-2.11927128	-0.45696822
H	-0.34730789	-3.09057319	-0.96375034
C	0.06544973	-2.32896448	1.00703172
H	1.10202259	-2.67802370	0.98419571
H	0.04705874	-1.35917685	1.51557048
C	-0.83357308	-3.31783250	1.69525647
C	-0.62487007	-4.69055729	1.53219705
C	-1.93984475	-2.88962446	2.43472374
C	-1.49876788	-5.61413182	2.09787958
H	0.23491140	-5.03203409	0.95879859

C	-2.81449989	-3.81149085	3.00313893
H	-2.11753630	-1.82317377	2.55459479
C	-2.59679555	-5.17660669	2.83513039
H	-1.32050188	-6.67802807	1.96537255
H	-3.66694213	-3.46232492	3.58027907
H	-3.27815585	-5.89742924	3.27909412
C	-1.77567862	-1.54266403	-0.47737645
O	-2.03250381	-0.45330863	0.05150381
N	-2.72702136	-2.25121433	-1.08819489
H	-2.54898603	-3.18181549	-1.44591987
C	-4.08720432	-1.77161785	-1.15179377
H	-4.37805113	-1.39665459	-0.16338369
C	-4.95569934	-2.96282985	-1.48519692
O	-4.54092411	-4.05290287	-1.80289015
O	-6.25703058	-2.65851722	-1.40746041
H	-6.74778490	-3.46015210	-1.65806660
C	-4.27532419	-0.62159602	-2.19247692
H	-3.33244226	-0.52529320	-2.73897601
H	-5.03552410	-0.91774024	-2.92418926
C	-4.66375974	0.70035675	-1.58326064
C	-5.97823439	0.91543987	-1.16030552
C	-3.72748444	1.72524447	-1.43225608
C	-6.35206288	2.13520004	-0.60332651
H	-6.71233368	0.12004852	-1.27401857
C	-4.10001077	2.94961313	-0.88560944
H	-2.69489472	1.56155329	-1.73044488
C	-5.41401074	3.15618822	-0.47200384
H	-7.37978219	2.29137836	-0.28481941
H	-3.36539289	3.74324128	-0.78310262
H	-5.70482423	4.11710084	-0.05493274
N	2.14868039	3.43115683	0.23866327
H	2.12806498	3.25877971	-0.76523593
C	0.78769665	3.32116437	0.74278859
H	0.82080996	3.30433378	1.83759410
C	-0.00550653	4.54395539	0.27290138
C	0.10662075	2.04738970	0.25967661
H	0.60853167	5.42239741	0.50270141
H	-0.08845211	4.50495247	-0.82158118
C	-1.36824061	4.71204884	0.86446266
O	0.14141936	1.70006304	-0.91204259
N	-2.16320506	5.76564855	0.46940932

C	-2.01801485	3.96655558	1.81844195
C	-3.26561656	5.65910114	1.17160785
N	-3.22694274	4.58896607	2.00605893
H	-1.74834960	3.06172527	2.33987378
H	-4.11658284	6.32582918	1.11928655
H	-3.97012001	4.26622120	2.60512382
O	-0.48730477	1.34282862	1.21230601
H	2.72614953	2.69662033	0.64205356
H	-1.01925468	0.60961822	0.78133047

The optimized atomic coordinates of the Dimer 3

H	-6.20644145	-2.28096201	-1.96053191
C	-5.32543885	-1.78236253	2.78014828
C	-4.31069209	-1.74171044	1.82274940
C	-4.61677522	-1.65481760	0.46377903
C	-5.94872922	-1.59718599	0.07870543
C	-6.97070161	-1.65472156	1.04202438
C	-6.66382157	-1.74563468	2.39712357
H	-5.06848872	-1.84914646	3.83399336
H	-3.27080470	-1.77865403	2.13672015
H	-3.82190174	-1.62556115	-0.27589897
H	-7.45244246	-1.78842261	3.14419129
C	-6.54205931	-1.46553314	-1.30518307
C	-8.02626708	-1.53928746	-1.03052859
C	-9.08571402	-1.49975615	-1.92468262
C	-10.38994998	-1.54522575	-1.42857025
C	-10.62376531	-1.62549843	-0.05509701
C	-9.56240339	-1.66353690	0.84649561
C	-8.26139207	-1.61892009	0.35318722
H	-8.90960520	-1.44055290	-2.99639678
H	-11.22990223	-1.51944742	-2.11747756
H	-11.64518334	-1.66008815	0.31454452
H	-9.74964128	-1.72754054	1.91541542
C	-6.19898402	-0.13402924	-1.98200646
H	-6.68603452	-0.07290755	-2.96007291
H	-6.52074950	0.70645823	-1.36219765
O	-4.79704390	-0.04485033	-2.21446043
C	-4.11849613	0.84033015	-1.44058478
O	-4.63015249	1.65226835	-0.69131730
N	-2.78988897	0.68493305	-1.60980118
H	-2.40138904	0.02787268	-2.27491025

C	-1.86389022	1.48283158	-0.85911327
H	-2.16795869	2.53595670	-0.91077322
C	-1.81437290	1.06164182	0.63760571
H	-2.83352472	0.76660032	0.90761200
H	-1.16712256	0.18344874	0.74201599
C	-1.36740083	2.17425251	1.54673272
C	-2.23506102	3.24614550	1.78900354
C	-0.09445047	2.18888832	2.12742716
C	-1.84004392	4.30862591	2.59474392
H	-3.22509888	3.23078509	1.33630123
C	0.30012162	3.25641918	2.93400599
H	0.59159879	1.37025082	1.91965325
C	-0.56981195	4.31736229	3.16972969
H	-2.52558805	5.13226044	2.77672510
H	1.29182219	3.25566877	3.38035073
H	-0.26115039	5.14808878	3.79891480
C	-0.49340318	1.29708606	-1.50190768
O	-0.27930989	0.41646953	-2.32432078
N	0.46640657	2.14691506	-1.06458635
H	0.25569739	2.69778426	-0.24124501
C	1.85633182	1.84093024	-1.30784004
H	2.04408745	1.80830051	-2.38469377
C	2.24438839	0.48315604	-0.72305434
O	1.79332056	0.06836069	0.33302829
O	3.16652448	-0.11022435	-1.44215409
H	3.59369895	-0.84798710	-0.88853835
C	2.71908964	2.93495074	-0.64738307
H	2.49029068	3.88601305	-1.14309468
H	2.39882433	3.02843661	0.39879451
C	4.19455966	2.64299004	-0.69882480
C	4.83165833	2.03904536	0.38739767
C	4.93719833	2.90303344	-1.85209837
C	6.17696754	1.68990376	0.31834778
H	4.25693069	1.81498858	1.28365739
C	6.28095167	2.55114424	-1.92603009
H	4.45168222	3.37184781	-2.70567585
C	6.90670636	1.93849768	-0.84203522
H	6.65218126	1.20637021	1.16857168
H	6.84175461	2.74786179	-2.83593379
H	7.94493958	1.62653969	-0.91384459
N	8.97441719	-1.06479467	-1.23457980

H	8.94267629	-1.63110825	-2.08081038
C	8.18142080	-1.72946770	-0.20886602
H	8.28121108	-1.16711974	0.72575017
C	6.71453659	-1.73620669	-0.64596226
C	8.66452459	-3.15748548	0.01057134
H	6.49608894	-0.74217821	-1.05136365
H	6.59707496	-2.45048628	-1.47260477
C	5.72918495	-2.03084052	0.43697026
O	8.83203512	-3.95606908	-0.88013709
N	4.39365499	-1.76525884	0.24141671
C	5.90468394	-2.51678443	1.70773775
C	3.77306156	-2.06949182	1.35807346
N	4.64962270	-2.53502237	2.27351263
H	6.78453066	-2.84290731	2.24043834
H	2.71717217	-1.92166929	1.53484815
H	4.42629129	-2.83196932	3.21032994
O	8.92667551	-3.44567113	1.30245553
H	9.95139010	-1.03683984	-0.95243249
H	9.22663275	-4.37208937	1.31642954

The optimized atomic coordinates of the Trimer 1

H	10.47909386	4.44370658	-2.18134784
C	9.40557277	4.28213002	2.54119447
C	8.77846037	3.45917081	1.60448410
C	9.26858450	3.36097594	0.30129097
C	10.39895933	4.08701409	-0.04668577
C	11.02126299	4.92727778	0.89273906
C	10.52865189	5.02744962	2.19113173
H	9.01015168	4.34620652	3.55144152
H	7.89830488	2.88921286	1.89052737
H	8.77310679	2.72067110	-0.42277342
H	11.00901450	5.67583320	2.91965236
C	11.14237859	4.13385834	-1.36205323
C	12.21755563	5.16064889	-1.09142729
C	13.19838842	5.64708866	-1.94278996
C	14.11687359	6.57735517	-1.45272964
C	14.05094440	7.00808582	-0.12698012
C	13.06802671	6.52097096	0.73174124
C	12.15110791	5.59388243	0.24420869
H	13.25218738	5.31767699	-2.97798927
H	14.88833348	6.97060721	-2.10920533

H	14.77358938	7.73284761	0.23859503
H	13.01982676	6.86101206	1.76313951
C	11.77671992	2.79218936	-1.74202971
H	12.34377182	2.89348534	-2.67255346
H	12.44080340	2.44099369	-0.94841234
O	10.76314472	1.82084406	-1.98072232
C	10.65219102	0.83154178	-1.05827617
O	11.43723769	0.62741702	-0.15035629
N	9.53588240	0.10955396	-1.28387071
H	8.92647855	0.28874993	-2.07221818
C	9.17642542	-0.96908988	-0.40960801
H	10.04951745	-1.61465784	-0.25070904
C	8.68723280	-0.45764777	0.97585718
H	9.28507736	0.43058095	1.20497869
H	7.63833238	-0.15063437	0.89419990
C	8.86823217	-1.47055848	2.07421118
C	10.15985001	-1.75887275	2.53154588
C	7.78451214	-2.15992350	2.62919534
C	10.36358817	-2.71459089	3.52124046
H	11.00111885	-1.21808013	2.10118461
C	7.99115199	-3.11907610	3.62060449
H	6.78228176	-1.95577534	2.25837751
C	9.27912343	-3.39902145	4.06863236
H	11.37168189	-2.92519823	3.86883243
H	7.14008988	-3.64709034	4.04438323
H	9.43906620	-4.14610443	4.84161020
C	8.06134545	-1.74714463	-1.10033739
O	7.47702724	-1.30605284	-2.08099269
N	7.74075310	-2.92970200	-0.52189422
H	8.12575209	-3.11199195	0.39709281
C	6.47695389	-3.55458201	-0.83458779
H	6.44235528	-3.80953488	-1.89747055
C	5.30260141	-2.62354683	-0.53722781
O	5.28916090	-1.86511644	0.41936539
O	4.30609105	-2.81497474	-1.36898471
H	3.46700130	-2.40752746	-0.97054087
C	6.33478736	-4.82653057	0.02605907
H	7.12972758	-5.52123279	-0.27042907
H	6.52135366	-4.54616819	1.07115338
C	4.98291765	-5.47860280	-0.08707489
C	3.98946642	-5.21272249	0.85773911

C	4.67491470	-6.30661618	-1.16839552
C	2.71429283	-5.75328834	0.72089191
H	4.21346116	-4.55439102	1.69447324
C	3.39993672	-6.84397409	-1.31169771
H	5.43951169	-6.52024684	-1.91262498
C	2.41183538	-6.56698864	-0.36882472
H	1.95057338	-5.52429493	1.46031443
H	3.17286746	-7.47641573	-2.16576915
H	1.40691632	-6.95918360	-0.49947062
N	-0.99535444	-5.40755092	-1.54233750
H	-1.14209972	-5.01546551	-2.47068098
C	-0.85019605	-4.31324803	-0.59549751
H	-0.76616439	-4.72980513	0.41412646
C	0.42526246	-3.53490799	-0.93753246
C	-2.03000960	-3.35544586	-0.63306627
H	1.21135224	-4.27561897	-1.12163733
H	0.26430120	-3.00018743	-1.88378694
C	0.90753884	-2.58046068	0.10608323
O	-2.44589166	-2.91411157	-1.70094802
N	2.15379083	-2.00989381	-0.01986143
C	0.33695248	-2.14596480	1.27654350
C	2.34518990	-1.26096553	1.04343335
N	1.26560145	-1.30812555	1.85254010
H	-0.61947717	-2.34968542	1.73196759
H	3.25490283	-0.71846623	1.25752841
H	1.15827238	-0.81744109	2.72651133
O	-2.53739884	-3.05312242	0.54122082
H	-1.83111491	-5.94544059	-1.32665991
H	-3.19327150	-2.29320157	0.44420857
H	-13.41112191	3.71815699	-0.76679873
C	-13.54242602	-1.10111472	-1.27065453
C	-12.42066000	-0.34447682	-1.61243887
C	-12.32718269	0.99736705	-1.24003562
C	-13.36349749	1.56663136	-0.51381874
C	-14.50006317	0.80835702	-0.18335144
C	-14.59367335	-0.52916330	-0.55830804
H	-13.59897210	-2.14439226	-1.56951033
H	-11.61307159	-0.80317440	-2.17705956
H	-11.45439568	1.58285340	-1.51494170
H	-15.47124153	-1.11802382	-0.30357859
C	-13.49494580	2.96966399	0.03302892

C	-14.87704549	2.95183851	0.64439919
C	-15.56668098	3.97436425	1.27852255
C	-16.83188033	3.70922304	1.80573428
C	-17.39134996	2.43516250	1.69916806
C	-16.70115434	1.40516284	1.06394428
C	-15.43991181	1.66839396	0.53685935
H	-15.13676770	4.97006426	1.36109460
H	-17.38630600	4.50139590	2.30144555
H	-18.37741694	2.24534041	2.11470565
H	-17.14255707	0.41513218	0.98213648
C	-12.45264649	3.29547685	1.10658993
H	-12.62289107	4.30000861	1.50596400
H	-12.49760613	2.56812686	1.92070887
O	-11.14498580	3.29504729	0.53924895
C	-10.32683152	2.28447567	0.92096491
O	-10.56896932	1.46961572	1.79005902
N	-9.18536100	2.31394093	0.19526652
H	-9.06674740	2.96493059	-0.56994775
C	-8.22769142	1.24953846	0.30346374
H	-8.05034285	1.04118659	1.36485690
C	-8.74061605	-0.04633975	-0.37661109
H	-9.71809512	-0.25925723	0.07053657
H	-8.89869733	0.17487074	-1.43870467
C	-7.81578895	-1.21996740	-0.20891677
C	-7.67681764	-1.83031389	1.04299166
C	-7.04945250	-1.70013941	-1.27467743
C	-6.79120702	-2.88879933	1.22383412
H	-8.28029328	-1.47576177	1.87681724
C	-6.17326711	-2.76878189	-1.10022697
H	-7.13830729	-1.22754830	-2.25050549
C	-6.03849847	-3.36298230	0.15141163
H	-6.69494677	-3.35055311	2.20295499
H	-5.58184640	-3.12585292	-1.93936455
H	-5.34548631	-4.18776936	0.29430055
C	-6.95155434	1.71500282	-0.38356905
O	-6.98544407	2.48491883	-1.33428401
N	-5.81283999	1.17345422	0.10870494
H	-5.87936562	0.46685342	0.83096306
C	-4.56309105	1.22253435	-0.61781650
H	-4.76508324	1.60131863	-1.62190839
C	-4.03254286	-0.19251690	-0.69080138

O	-3.91637730	-0.84771745	0.34422887
O	-3.70993463	-0.59808479	-1.88565168
H	-3.29169519	-1.50905092	-1.83595030
C	-3.51318027	2.10510670	0.08770860
H	-3.90204367	3.12880909	0.09727406
H	-3.43921078	1.76584850	1.12727737
C	-2.16917397	2.03375303	-0.58966725
C	-1.23502913	1.06151608	-0.21674231
C	-1.86088331	2.88539457	-1.65301943
C	-0.02566701	0.93663841	-0.89412727
H	-1.46567104	0.38432852	0.60309334
C	-0.64874640	2.77000605	-2.32707898
H	-2.57936335	3.64528994	-1.95389602
C	0.27018314	1.79308923	-1.95140628
H	0.68344686	0.16690725	-0.60319447
H	-0.42268155	3.44313962	-3.14976660
H	1.21448177	1.69821659	-2.48047377

The optimized atomic coordinates of the Trimer 2

H	-4.72358800	-1.50531400	-0.38872800
C	-7.15889800	1.92507500	2.00031400
C	-5.79300500	1.74321000	2.15067500
C	-5.07930700	0.92017400	1.28751400
C	-5.76392700	0.28792200	0.27062000
C	-7.14057300	0.46387100	0.12215100
C	-7.84618400	1.28325500	0.98214700
H	-7.68892500	2.56383600	2.67942300
H	-5.27735800	2.24639200	2.94517700
H	-4.02527700	0.78863000	1.41547100
H	-8.90410600	1.41978700	0.87117400
C	-5.23228000	-0.64710500	-0.81029900
C	-6.50530900	-1.02624300	-1.55651900
C	-6.66761700	-1.88169000	-2.62335400
C	-7.93721700	-2.05795800	-3.16036500
C	-9.02405600	-1.38377100	-2.62839400
C	-8.86463600	-0.52574800	-1.54979600
C	-7.60256000	-0.35218700	-1.01725100
H	-5.83097000	-2.41091700	-3.03771500
H	-8.07657200	-2.72189100	-3.99054500

H	-9.99820700	-1.52904100	-3.05199300
H	-9.71046100	-0.01063300	-1.13866500
C	-4.27913000	0.10137200	-1.76393900
H	-3.98752900	-0.51782200	-2.61809500
H	-4.74039600	1.02083000	-2.12755200
O	-3.10555900	0.41026200	-0.98748800
C	-2.21818300	1.27893700	-1.52600500
O	-2.32967500	1.77801300	-2.63869700
N	-1.17380100	1.47772800	-0.66479700
H	-1.31571200	1.20174800	0.31083500
C	-0.26162200	2.57565900	-0.91938300
H	-0.23789500	2.74285000	-1.99828000
C	-0.74201200	3.87388000	-0.20079200
H	-1.73942300	4.08807700	-0.56343500
H	-0.80025500	3.65753700	0.85732900
C	0.18149600	5.04253400	-0.47236900
C	0.03659600	5.79610400	-1.62952000
C	1.20353600	5.36141400	0.41205700
C	0.89464400	6.84787800	-1.89872500
H	-0.75601500	5.56454700	-2.31577100
C	2.06154200	6.41545700	0.14575100
H	1.32894400	4.77662400	1.30174100
C	1.90973300	7.16063000	-1.01076500
H	0.76686100	7.42463600	-2.79353500
H	2.84012900	6.65676700	0.84251300
H	2.57073600	7.97954600	-1.21492700
C	1.12199200	2.20792600	-0.37817500
O	1.31101900	2.05668800	0.83641100
N	2.10228900	2.10986600	-1.30637400
H	1.82555000	2.29717200	-2.26012700
C	3.53181700	1.84475000	-1.09944600
H	4.03361500	2.18729600	-2.00581800
C	3.81080500	0.33873800	-0.97766300
O	3.72320400	-0.27258600	0.08968500
O	4.11310900	-0.22485500	-2.12834400
H	4.31070600	-1.21260100	-2.00900600
C	4.11676800	2.58850800	0.13541300
H	3.81970300	3.62682600	0.06529200
H	3.67338700	2.16523300	1.02061300
C	5.62730000	2.46982100	0.15478700
C	6.25350600	1.49231200	0.91480100

C	6.40905600	3.33489400	-0.60017500
C	7.63408600	1.38356200	0.92122900
H	5.65616000	0.81724500	1.49405900
C	7.78745700	3.22619400	-0.59764200
H	5.93668600	4.10313400	-1.18311000
C	8.40418900	2.24844600	0.16488700
H	8.10543600	0.62811000	1.51856600
H	8.37803700	3.90333700	-1.18260000
H	9.47287700	2.16546800	0.17142300
N	6.18508000	-5.06229300	0.22198100
H	6.02249600	-4.67804500	1.14923800
C	5.02339800	-4.86421600	-0.64198500
H	5.29632200	-5.20075100	-1.64689300
C	3.83161600	-5.68376800	-0.11341300
C	4.61948500	-3.39303100	-0.76923800
H	4.18301600	-6.71202800	0.03073200
H	3.54334200	-5.30691600	0.87397100
C	2.62709500	-5.65638300	-1.01274000
O	4.65322200	-2.77394400	-1.83714300
N	1.33844300	-5.77976000	-0.51299700
C	2.57818900	-5.55982600	-2.38623300
C	0.54233800	-5.77464800	-1.56490900
N	1.24241000	-5.63789700	-2.72324400
H	3.34482000	-5.42957800	-3.13395800
H	-0.53532800	-5.85990000	-1.54004500
H	0.85668800	-5.56650200	-3.65325100
O	4.27900700	-2.84175800	0.38123200
H	7.01643100	-4.62197900	-0.16210200
H	4.06662900	-1.86005200	0.26982900
N	-2.77238700	-0.45615200	4.37934800
H	-3.04400100	-1.19276000	3.72992600
C	-1.34879700	-0.19923500	4.28049000
H	-1.05953300	0.46704200	5.10661000
C	-0.54256700	-1.51072700	4.46927100
C	-0.90932700	0.54804200	3.01172300
H	0.51163300	-1.26583500	4.62358800
H	-0.91753900	-1.96494800	5.39432900
C	-0.65575100	-2.48591200	3.33110700
O	-1.67341200	0.91629600	2.12422600
N	-1.87281000	-2.90379600	2.82101200
C	0.36639900	-3.10345300	2.64416100

C	-1.58502600	-3.74453800	1.84514600
N	-0.24036200	-3.90474400	1.69972900
H	1.43969900	-3.01731000	2.71848200
H	-2.30619600	-4.25702000	1.22357900
H	0.24387300	-4.48129700	1.01702300
O	0.39849700	0.80195200	2.99009500
H	-3.31722800	0.37330300	4.16495200
H	0.64104700	1.29521300	2.16698700
