# Supplementary material for: Histidine modulates amyloid-like assembly of peptide nanomaterials and confers enzyme-like activity
Source: Nat Commun. 2023 Sep 19;14:5808. doi: 10.1038/s41467-023-41591-1 (PMC10509148; doi:10.1038/s41467-023-41591-1)
Supplement: Supplementary file 5 — Reporting Summary [file 41467_2023_41591_MOESM5_ESM.pdf]

nature portfolio

Corresponding author(s): Lizeng Gao

Last updated by author(s): Aug 20, 2023

Reporting Summary

Nature Portfolio wishes to improve the reproducibility of the work that we publish. This form provides structure for consistency and transparency in reporting. For further information on Nature Portfolio policies, see our Editorial Policies and the Editorial Policy Checklist.

Statistics

For all statistical analyses, confirm that the following items are present in the figure legend, table legend, main text, or Methods section.

n/a	Confirmed	
☐	☒	The exact sample size (*n*) for each experimental group/condition, given as a discrete number and unit of measurement
☐	☒	A statement on whether measurements were taken from distinct samples or whether the same sample was measured repeatedly
☐	☒	The statistical test(s) used AND whether they are one- or two-sided<br>*Only common tests should be described solely by name; describe more complex techniques in the Methods section.*
☐	☒	A description of all covariates tested
☐	☒	A description of any assumptions or corrections, such as tests of normality and adjustment for multiple comparisons
☐	☒	A full description of the statistical parameters including central tendency (e.g. means) or other basic estimates (e.g. regression coefficient) AND variation (e.g. standard deviation) or associated estimates of uncertainty (e.g. confidence intervals)
☐	☒	For null hypothesis testing, the test statistic (e.g. *F*, *t*, *r*) with confidence intervals, effect sizes, degrees of freedom and *P* value noted<br>*Give P values as exact values whenever suitable.*
☒	☐	For Bayesian analysis, information on the choice of priors and Markov chain Monte Carlo settings
☒	☐	For hierarchical and complex designs, identification of the appropriate level for tests and full reporting of outcomes
☒	☐	Estimates of effect sizes (e.g. Cohen's *d*, Pearson's *r*), indicating how they were calculated

Our web collection on statistics for biologists contains articles on many of the points above.

Software and code

Policy information about availability of computer code

Data collection	Cryo-EM and two-dimensional classification data were collected by Talos Arctica 200 kV FEG. DFT calculation was conducted using the Gaussian09 package. SAXS fitting was conducted using SaSView 5.0.6 package. The ROS levels of cells were analyzed using FlowJo_V10 software.
Data analysis	GraphPad Prism 7

For manuscripts utilizing custom algorithms or software that are central to the research but not yet described in published literature, software must be made available to editors and reviewers. We strongly encourage code deposition in a community repository (e.g. GitHub). See the Nature Portfolio guidelines for submitting code & software for further information.

Data

Policy information about availability of data

All manuscripts must include a data availability statement. This statement should provide the following information, where applicable:

- Accession codes, unique identifiers, or web links for publicly available datasets
- A description of any restrictions on data availability
- For clinical datasets or third party data, please ensure that the statement adheres to our policy

All data are available in the main text or Supplementary Materials. All electron microscope images are available upon request to the corresponding authors. Source data are provided with this paper.

Human research participants

Policy information about studies involving human research participants and Sex and Gender in Research.

Reporting on sex and gender	n/a
Population characteristics	n/a
Recruitment	n/a
Ethics oversight	n/a

Note that full information on the approval of the study protocol must also be provided in the manuscript.

Field-specific reporting

Please select the one below that is the best fit for your research. If you are not sure, read the appropriate sections before making your selection.

☒ Life sciences ☐ Behavioural & social sciences ☐ Ecological, evolutionary & environmental sciences

For a reference copy of the document with all sections, see nature.com/documents/nr-reporting-summary-flat.pdf

Life sciences study design

All studies must disclose on these points even when the disclosure is negative.

Sample size	The in vitro experiments were independently performed three times and standard number of replication is three in all in vitro studies. At least three mice were used for in vivo studies and details regarding sample size of in vivo studies are provided in the methods section. All animal studies and the sample sizes were performed following the protocols approved by the Institutional Animal Care and Use and Committee of the Institute of Biophysics, Chinese Academy of Sciences.
Data exclusions	No data were excluded.
Replication	Each experiments were repeated at least three times and experimental findings were reproducible.
Randomization	Samples were randomly allocated to corresponding experimental groups.
Blinding	The experiments and results analyses were performed by multiple researchers, who had minimal information of sample identification. However, samples were not formally blinded.

Reporting for specific materials, systems and methods

We require information from authors about some types of materials, experimental systems and methods used in many studies. Here, indicate whether each material, system or method listed is relevant to your study. If you are not sure if a list item applies to your research, read the appropriate section before selecting a response.

Materials & experimental systems

n/a	Involved in the study	
☒	☐	Antibodies
☐	☒	Eukaryotic cell lines
☒	☐	Palaeontology and archaeology
☐	☒	Animals and other organisms
☒	☐	Clinical data
☒	☐	Dual use research of concern

Methods

n/a	Involved in the study	
☒	☐	ChIP-seq
☐	☒	Flow cytometry
☒	☐	MRI-based neuroimaging

Eukaryotic cell lines

Policy information about cell lines and Sex and Gender in Research

Cell line source(s)	The HT-22 cell line (SCC129) was obtained from Sigma Aldrich/Merck. The PC-12 cell line (CRL-1721) was obtained from ATCC.

Authentication	The cell line was not independently authenticated.
Mycoplasma contamination	All cell lines were tested negative for mycoplasma contamination.
Commonly misidentified lines (See ICLAC register)	No commonly misidentified cell lines were used.

Animals and other research organisms

Policy information about studies involving animals; ARRIVE guidelines recommended for reporting animal research, and Sex and Gender in Research

Laboratory animals	SD rats, male, 6-8 weeks old
Wild animals	The study did not involve wild animals.
Reporting on sex	The study did not involve female animals. The reason is female rats do not easily construct a AD model due to the influence of estrogen.
Field-collected samples	The study did not involve samples collected from the field.
Ethics oversight	All animal studies were performed following the protocols approved by the Institutional Animal Care and Use and Committee of the Institute of Biophysics, Chinese Academy of Sciences.

Note that full information on the approval of the study protocol must also be provided in the manuscript.

Flow Cytometry

Plots

Confirm that:

☒ The axis labels state the marker and fluorochrome used (e.g. CD4-FITC).

☒ The axis scales are clearly visible. Include numbers along axes only for bottom left plot of group (a 'group' is an analysis of identical markers).

☒ All plots are contour plots with outliers or pseudocolor plots.

☒ A numerical value for number of cells or percentage (with statistics) is provided.

Methodology

Sample preparation	HT-22 could be adherently grown in RPMI 1640 culture medium containing 10 % fetal calf serum and 1% penicillin and streptomycin. In animal experiments, the right side of fresh hippocampal tissue was prepared into a single cell suspension. PC-12 cells (CRL-1721, obtained from ATCC) were seeded into 12-well plates were treated with 50 ng/mL Nerve Growth Factor (NGF) to induce neuronal differentiation for 10 d (RPMI 1640 medium supplemented with 10% horse serum (HS), 5% FBS, and 1% PS) and detected consistent with HT-22.
Instrument	The data was collected by Backman Coulter CYTOFLEX.
Software	The data was analyzed by FlowJo_V10.
Cell population abundance	We collected 10 000 cells per sample (HT-22 cell lines, PC-12 and histocyte suspension in the hippocampus) for testing.
Gating strategy	HT-22 cells were gated to exclude aggregates and select singlets. PC-12 cells were gated as singlets, then BODIPY, MitoSox and DCFH-DA probes MFI of cells were anaylzed.

☒ Tick this box to confirm that a figure exemplifying the gating strategy is provided in the Supplementary Information.
